# Can black soldier fly larvae (*Hermetia illucens*) be utilized by ruminants? a meta-analysis of performance, digestibility, and rumen fermentation responses

**DOI:** 10.1186/s12917-026-05586-6

**Published:** 2026-05-28

**Authors:** Yulianri Rizki Yanza, Nahrowi, Muhammad Dzaky Alifian, Agung Irawan

**Affiliations:** 1 Faculty of Animal Husbandry, Universitas Padjadjaran, Sumedang, 45363 Indonesia; 2 Department of Animal Nutrition and Feed Technology, Faculty of Animal Science, IPB University, Bogor, 16680 Indonesia; 3 Center for tropical Animal studies (Centras), IPB University, Kampus Baranangsiang, Bogor, 16680 Indonesia; 4 Research Center for Animal Husbandry, National Research and Innovation Agency (BRIN), Bogor, 16915 Indonesia; 5 Vocational School, Universitas Sebelas Maret, Surakarta, 57126 Indonesia

**Keywords:** Feed ingredient, Insect, Livestock, Protein alternative, Responsible consumption and production

## Abstract

**Background:**

This meta-analysis examined the effects of dietary black soldier fly (BSF) on ruminant production, digestibility, and rumen fermentation parameters, and key moderating variables that influenced the animal responses. In ruminants, a total of 12 studies were identified through Scopus, Web of Science, and PubMed and were analyzed using mixed model meta-analysis using the metafor package in R software.

**Results:**

The results indicated that BSF inclusion did not affect dry matter intake (DMI), average daily gain (ADG), feed efficiency (FCR), and digestibility outcomes. However, crude protein intake decreased (RMD = − 3.54%; *P* = 0.006) with BSF inclusion. While milk yield and dairy efficiency were unaffected, BSF inclusion exhibited a positive effect on milk fat percentage (+ 3.16%; *P* = 0.001). Rumen fermentation was enhanced by BSF inclusion, as indicated by higher total VFA production (+ 7.69%; *P* = 0.024). Animal species and inclusion levels were significant moderators of DMI and DMD, in which BSF inclusion in goats resulted in improved ADG (*P* = 0.019) and DMI (*P* < 0.01), while no effect was found on sheep. Beef and dairy cattle displayed improved DM digestibility (*P* = 0.04). No major effects were detected for BSF type or country of study, and publication bias was not evident for the main production outcomes.

**Conclusions:**

Our meta-analysis suggests that BSF-derived ingredients can be incorporated into ruminant diets as a partial replacement for soybean meal without compromising overall animal performance while providing specific benefits for milk quality and rumen fermentation. However, additional studies on growing ruminants, particularly steers and lambs, are warranted, as current evidence on these classes is limited.

## Introduction

The global livestock industry faces significant challenges in maintaining animal productivity while reducing the environmental impact of conventional feed resources. Feed costs represent over 70% of the total expenses in livestock production, with an increasing reliance on protein ingredients such as soybean meal and fishmeal [1]. These ingredients not only compete with human food and industrial uses but also contribute to environmental concerns, including deforestation, greenhouse gas (GHG) emissions, and depletion of marine resources [2]. Consequently, identifying and adopting alternative sustainable feed ingredients has become essential for the ruminant sector to improve feed efficiency and animal performance and reduce environmental impact without compromising animal welfare or product quality.

Insects, particularly the black soldier fly (*Hermetia illucens*, BSF), have emerged as a promising alternative to conventional feed sources because of their efficiency in converting organic waste into high-quality proteins and lipids. BSF larvae can transform low-value organic substrates, such as food scraps, agricultural residues, and manure, into nutrient-dense biomass with minimal land, water, and GHG emissions [3, 4]. These characteristics position BSF as an ideal component of circular bioeconomy models that prioritize nutrient recycling and waste valorization in sustainable livestock production.

Although BSF meal has been extensively studied in monogastric diets (poultry, swine, and aquaculture), its application in ruminant nutrition remains underexplored. Ruminants have a complex microbial ecosystem within the rumen that plays a crucial role in breaking down fibrous plant materials and synthesizing microbial proteins to support growth and productivity [5]. The inclusion of BSF in ruminant diets may not only offer high-quality nutrition, but also influence microbial ecology, fermentation pathways, and metabolic efficiency within the rumen [6]. Understanding these interactions is critical for determining whether BSF enhances or disrupts ruminal fermentation and overall animal performance.

BSF larvae are particularly valuable because of their high crude protein (CP) content (40–55% DM) and moderate lipid levels (25–35% DM), with an amino acid profile comparable to that of fishmeal [7]. In addition to essential nutrients, BSF contains bioactive compounds, such as medium-chain fatty acids (MCFA), particularly lauric acid and myristic acid, as well as chitin, which are known for their functional roles in modulating rumen fermentation and microbial activity [8, 9]. Lauric acid is best known to exert antimicrobial properties that can inhibit the growth of gram-positive bacteria, such as *Streptococcus bovis* and *Clostridium* spp., which are often associated with inefficient fermentation and lactic acidosis [10, 11]. By modulating rumen microbial populations, BSF-derived lipids could enhance rumen fermentation efficiency by increasing volatile fatty acid (VFA) production [12, 13].

Furthermore, chitin in the exoskeleton of BSF larvae is a fibrous polysaccharide that can be partially degraded by microbes in the rumen, contributing to fermentable substrate especially N-acetylglucosamine release [14]. Chitin and its primary derivative, chitosan, may also potentially modulate rumen biohydrogenation and lipid metabolism, further optimizing rumen fermentation that would contribute to improve feed efficiency [15]. Studies have demonstrated that BSF inclusion can improve rumen fermentation by increasing VFA production, reducing rumen ammonia (NH_3_-N) concentration, and improving organic matter digestibility [16, 17].

A number of in vitro studies have shown that replacing soybean meal with defatted BSF leads to higher total gas and VFA production while decreasing CH₄ emissions [18]. In vivo studies have also demonstrated that BSF supplementation enhances growth rates and feed conversion efficiency in cattle consuming low-quality forage [9, 15]. These findings suggest that BSF can serve as a nutrient-dense protein source and functional additive that improves rumen efficiency and mitigates methane emissions. Moreover, several studies have reported enhanced nitrogen utilization efficiency, blood metabolite level, and immune function in goats and sheep [15, 19]. These results highlight the promising role of BSF derived products in enhancing fermentation efficiency and reducing nitrogen excretion, which aligns with the sustainability goals of minimizing environmental pollution from livestock.

Despite these promising outcomes, the literature reveals significant variability in the effects of BSF on ruminant performance, rumen fermentation, and metabolism. Differences in BSF processing (e.g., full-fat vs. defatted meals), inclusion levels, animal species, and diet composition contribute to the inconsistencies across studies. Some studies have shown improved digestibility and VFA production with moderate BSF inclusion, while others have reported neutral or adverse effects at higher inclusion rates [16, 20]. These discrepancies underscore the need for a meta-analysis [21, 22] to synthesize the existing data, identify patterns, and determine the optimal conditions for BSF larvae supplementation .

A meta-analysis synthesizes data from various studies is powerful statistical method to elucidate the impact of BSF on ruminant animals, encompassing both live animal trials and laboratory experiments. Consequently, the present study was designed to determine the dose–response relationship between BSF inclusion levels and indicators of digestibility and feed efficiency; to assess the effects of BSFL on rumen fermentation end products, including total VFA, acetate-to-propionate ratio, and NH_3_-N; and to evaluate the implications for animal productivity, such as weight gain, milk yield, and nutrient utilization.

## Materials and methods

### Articles search

To obtain relevant publications, scientific databases including Scopus (https://www.scopus.com), PubMed (http://www.ncbi.nlm.nih.gov/pubmed), and Web of Science (https://www.webofscience.com/) were used as the main platforms considering their high reputation and coverage for most journal publications. The article search was conducted in September 2025. In addition, a manual search was also conducted across animal and veterinary-related peer-reviewed journals to identify suitable articles that might not have been indexed in the above databases (e.g., in press or accepted articles). The search strategy utilized keywords “black soldier fly” or “*Hermetia illucens*” AND “Ruminants”, “Sheep”, “Lamb”, “Goat”, “Cattle”, “Dairy cows” on each database. The titles from each database were imported into Microsoft Excel to facilitate the screening and selection process.

### Inclusion criteria and study selection

**Fig. 1:**
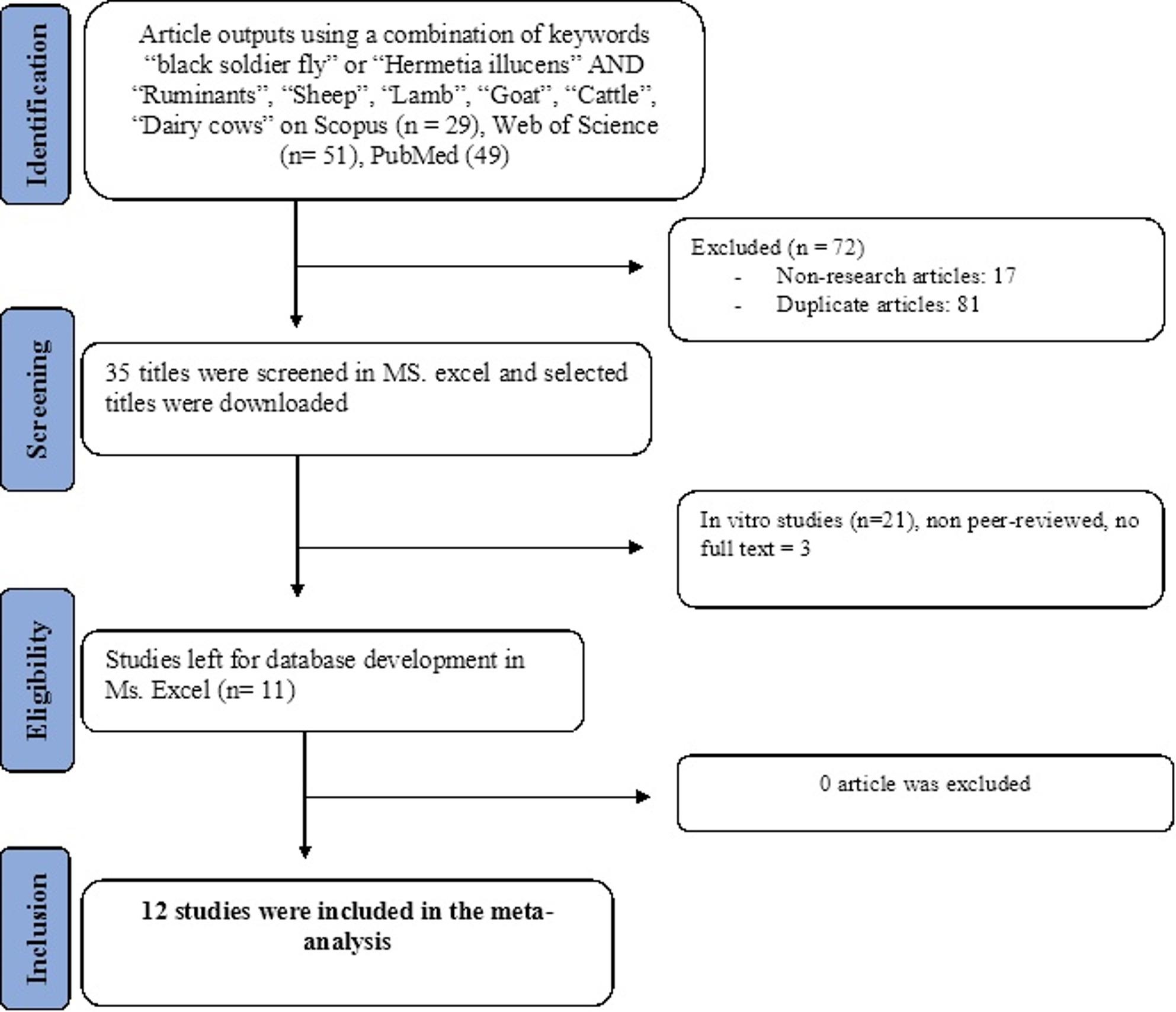
Flowchart of article selection based on PRISMA protocol

Articles collected in the spreadsheet were assessed for their eligibility for the meta-analysis according to the following criteria: (1) published in peer-reviewed journals covered in Scopus, Web of Science, or PubMed databases; (2) reported original research article reporting the use of BSF and their co-products in the vivo experiment; (3) employed appropriate animal randomization (e.g., randomized control trial); (4) reported the use of BSF and their co-products as dietary substitute or supplement as a means of testing their suitability as ruminant feed ingredient which were compared to basal diet or control groups; (5) presented the quantitative data (mean value and their variance) of the production performance (average daily gain or ADG and dry matter intake or DMI) and other relevant parameters such as nutrient utilization, and rumen fermentation; (6) reported detailed information of replicates and animal specifics (breed, number, body weight), experimental settings, diets, methods, and statistical analysis; and (7) reported the adherence to the ethical committee for animal research (IACUC/ACUP). Representative summary of the selection process following the PRISMA protocol [23] is shown in Fig. 1.

### Data extraction

Relevant information from the selected studies (Table 1) was systematically extracted and coded into an excel spreadsheet. This includes general information of the article (e.g., authors, country, journal, publication year), and detailed of the experimental setting such as number of animals (replicates) used in the trial, animal specificity (breed, physiological phase, sex, experimental period (days), types of the diets and their chemical composition, types of BSF products (meal or oil), and the inclusion levels. The quantitative data related to outcomes were organized in the column after the general information and experimental settings, which includes the mean and variance (SD or SEM). Some calculations were perform to obtain missing data such as ADG and FCR; they were calculated using the final and initial BW and was divided by experimental period (days; kg/d for ADG) and ADG/DMI for FCR. The variance was standardized into SEM; thus transformation was made from SD to SEM using the formula SEM = SD/sqrt(n; number of replicate). We utilized online platform of Web-Plot-Digitizer (https://apps.automeris.io/wpd/) to extract graphical data in the article to assure all data were properly extracted and included in the analysis [24, 25].

When a study involved multiple experiments, we treated each experiment as independent, nested within a study. Next to the SEM for each response variable, weighting factor (WF) was added. The WF value, also referred to as normalized inverse variance matrix was computed as WF = W1/W2, where W1 represents the reciprocal of the experiment’s SEM (1/SEM), and W2 is general average of all W1 values across the specific outcome [26]. In addition, several categorical variables were added to be included in the analyses. Table 1 summarizes the study characteristics included in this meta-analysis. 

**Table 1:** Lists of eligible studies included in the meta-analysis

Study	Authors	Year	Country	Animal	Purpose	Length (d)	BSF Type	Level (% DM)
1	Phowang et al. [27]	2025	Thailand	Beef	25 - 75% replace SBM	21	BSFM	0; 3.75; 7.5; 11.25
2	Lu et al. [5]	2024	Thailand	Goat	100% replace fullfat soybean	60	BSFM	0; 10
3	Odeon et al. [28]	2025	Argentina	Lamb	100% replace SBM	50	BSFM	0; 10
4	Fernandez-Mora et al. [29]	2025	USA	Sheep	25 and 50% replace SBM	21	BSFM	0; 2.5; 5
5	Braamhaar et al. [30]	2025	Kenya	Dairy cows	50% replace SBM	18	BSFM	0; 4.5; 8.9
6	Carrasco and Drewery [9]	2024	USA	Beef	100% replace SBM	14	BSFM	0; 3; 5.9
7	Fukuda et al. [15]	2022	USA	Beef	100% replace SBM	14	BSFM	0; 10.5
8	Nekrasov et al. [13]	2022	Russia	Dairy cows	Top-dressed supplement	176	BSFM	0; 0.02; 0.2
9	Lu et al. [31]	2025	Thailand	Goat	24 - 60% replace SBM	75	BSFM	0; 5; 10; 15
10	Rastello et al. [32]	2025	Italy	Dairy cows	100% replace fat	50	BSFO	0; 3.4
11	Rahman et al. [33]	2020	Indonesia	Sheep	100% replace SBM	84	BSFM	0; 2.5; 5
12	Prachumchai et al. [34]	2025	Thailand	Beef	Fat supplement	21	BSFO	0; 1; 2; 4

*BSFM *defatted black soldier fly larvae meal, *BSFO *black soldier fly oil, *DM *dry matter

### Publication bias assessment

**Fig. 2:**
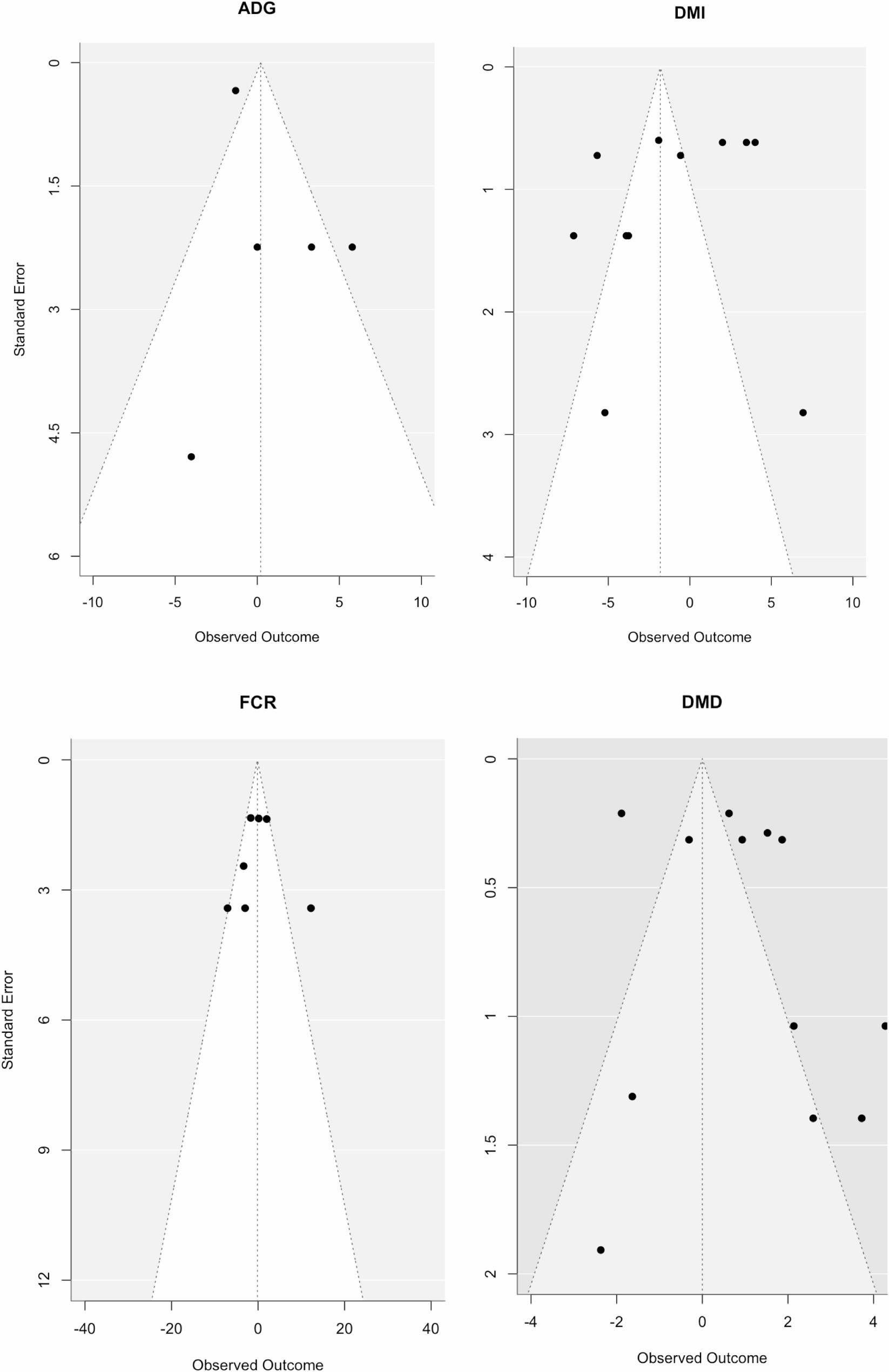
Funnel plot of publication bias assessment on the main parameters

Publication biases were assessed visually using funnel plots to inspect the symmetrical dataset for the main outcomes (e.g., production performance dataset; Fig. 2) and statistically using Egger’s test and Begg’s test [35], with the *P*-values reported for each outcome. In addition, a sensitivity analysis was also carried out to evaluate the robustness of the treatment effects by identifying potential outliers or influential data from a specific study. For this purpose, a leave-one-out approach was used [36].

### Meta-analysis

The meta-analysis was performed following the mixed-effect model in the RStudio environment (RStudio version 2024.4.2 + 764) using the “*metafor*” package [37]. In addition, meta-regression was also carried out using “*lme4*” and “*lmerTest*” packages [38]. Before meta-analysis and meta-regression, the dataset was assessed for potential outliers using visual and statistical approaches. For each outcome, Cook’s distance and studentized residuals were calculated using the “*influence*()” function. Datapoint was deemed an outlier and removed for those with Cook’s distance > 3, the mean value, and/or a studentized residual value greater than 3. This data driven approach, as previously suggested by Kebreab et al. [39] to be more robust for meta-analysis was used to avoid excessive data removal. Following this, a meta-analysis was performed using the clean dataset.

For the meta-analysis, effect size estimation was carried out for each outcome using a weighted relative mean difference (%RMD), considering the random effect of “study_id” and fixed effect of treatment groups, and was weighted using the WF value. This approach provides constant estimates of the magnitude of the effect size, regardless of the experimental units. The equations for calculating the % RMD and its variance are presented as follows:

$$\:\mathrm{R}\mathrm{M}\mathrm{D}=\left(\square\frac{\overset-YT\:-\:\:\overset-YC}{\overset-YC}\right)\:\times\:100$$

$$\:\mathrm{V}\mathrm{a}\mathrm{r}\left(\mathrm{R}\mathrm{M}\mathrm{D}\right)\approx\:100^2\times\:\left[\frac1{\left(\overset-YC\right)^2}\times\:var\left(\overset-YT\right)+\:\left(\frac{\overset-YT}{\left(\overset-YC\right)^2}\right)^2\times\:var\left(\overset-YC\right)\right]$$

and inverse-variance weighted = $\:\frac{\sum\:Yi\left(\frac{1}{{SE}_{i}^{2}}\right)}{\sum\:\left(\frac{1}{{SE}_{i}^{2}}\right)}$.

where $\:\stackrel{-}{Y}T$ = mean value of the BSF-related group, $\:\stackrel{-}{Y}C$ = mean value of the control, SE_1_ = SE of the BSF-related group, SE_2_ = SE of the control group, $\:var\left(\stackrel{-}{Y}T\right)$ = SEM^2^ of the treatment or control groups [37]. The RMD is plotted using a forest plot and is presented as a 95% confidence interval (95% CI). Heterogeneity value derived from different studies was computed using Cochran’s *Q* and I^2^ statistics [40] by employing Der Simonian–Laird (DL) estimator. To facilitate easy and direct interpretation, we used an intercept-free structure in the model. To facilitate the identification of moderator effects, subgroup meta-analysis was conducted. It is typical that for each study, multi-arm treatments shared a similar control group, causing a non-independent effect of the treatment. To handle this, we used a multilevel mixed-effects meta-analysis approach to calculate the effect size with the *rma.mv()* function in the metafor package in R, whereas the subgroup was declared as a moderator and study-specific effect was used as a random effect in the model [(i.e., mods = ~ 0 + covariates + (1 | study_id)]. The heterogeneity indicator was used as no heterogeneity (0% < *I*² ≤ 25%), low heterogeneity (25% < *I*² ≤ 50%), moderate heterogeneity (50% < *I*² ≤ 75%), or high heterogeneity (*I*² > 75%).

## Results and discussion

### Animal production and nutrient utilization

A total of 12 in vivo studies were identified to be eligible for meta-analysis, which were published between 2020 and 2025 (Table 1). Among them, 7 out of 12 studies were published recently in 2025, indicating that in vivo studies in ruminants are new and there is a rapidly growing interest in using BSF in ruminants. The geographical distribution of the studies was highly diverse, covering the Asian region (Thailand, Indonesia), North and South America (USA, Argentina), Africa (Kenya), and Europe (Italy, Russia), with Thailand contributing the highest number of studies. Across the dataset, the experiments involved several ruminant species, including beef cattle (4 studies), dairy cows (3 studies), goats (2 studies), and sheep (3 studies). The purposes of most studies were to examine the potential of defatted BSF larvae meal (BSFM) to partially or fully replace SBM with dietary inclusion rates ranging from 0.02% to 11.25% of DM. There were variabilities in the length of the experiments, ranging from 14 to 176 days, with five studies utilizing rumen fistulated animals (*in sacco*). Overall, the included studies show a growing global interest in utilizing BSF-derived ingredients, especially defatted BSFM (10 studies) and BSFO (2 studies), into ruminant nutrition.

Table 2 summarizes the descriptive statistics of the dataset from eligible studies included in the meta-analysis. Across 25–35 observations, the chemical composition of the diets showed quite homogenous ranges, especially the CP level (averaged 14.89%), while DM and NDF varied considerably due to the diets’ composition used across studies and animal species. The NDF content of the diets averaged 28.55±11.59 in cattle and 35.24±10.55 in small ruminants. The feed intake data are within the expected ranges for small and large ruminants. Data on DM, CP, and NDF digestibility showed moderate variations, yet all are within the expected ranges. The production performance data showed the average ADG of 0.13 kg/d for small ruminants, which aligns with typical performance values for growing small ruminants under controlled feeding trials. Other performance metrics, including feed conversion ratio (FCR), milk production data for dairy cows, and feed efficiency (milk yield/ DMI), were within the normal ranges, suggesting the reliability of the data. Rumen fermentation parameters demonstrated moderate variability across 8–28 observations. Total VFA averaged 97.92 mmol, and individual VFA proportions showed typical ruminal fermentation patterns, with acetate averaging 68.15%, propionate 18.70%, and butyrate 11.78%. The acetate-to-propionate (A/P) ratio averaged 3.12, which is consistent with forage-based diets.

**Table 2:** Descriptive statistics of the dataset

Items	Descriptive statistics				
	N	Average	SD	Min	Max
Chemical composition of diets					
DM	35	84.84	19.88	35.00	95.70
OM	25	93.47	1.54	90.70	95.00
CP	31	14.89	1.05	12.40	16.20
NDF	35	32.04	11.51	14.80	44.92
Feed intake (kg/d) and digestibility (%)					
DMI, kg/d	39	6.26	6.05	0.75	17.60
CPI, kg/d	19	1.11	0.90	0.21	2.68
NDFI, kg/d	17	4.07	2.27	0.71	7.45
DMD, %	32	66.00	4.54	60.20	77.60
CPD, %	25	67.39	4.01	62.10	74.98
NDFD, %	32	58.09	6.15	45.20	68.40
Production performance					
ADG, kg/d	20	0.13	0.05	0.07	0.23
FCR	20	8.23	2.81	5.80	12.76
Milk yield, kg	8	25.58	5.41	19.50	32.96
Milk yield/ DMI	3	1.34	0.01	1.33	1.34
ECM, kg	5	21.40	3.41	17.30	24.40
ECM/ DMI	3	1.38	0.03	1.35	1.41
Milk fat, %	8	3.79	0.11	3.66	3.96
Milk protein, %	8	3.21	0.19	3.04	3.47
Milk lactose, %	8	4.93	0.25	4.67	5.28
Rumen fermentation					
N-NH_3_, mg/dL	4	13.65	0.90	12.60	14.70
BUN	18	13.17	2.39	8.50	16.57
Protozoa	8	1.34	0.39	1.00	1.88
pH	24	6.83	0.17	6.51	7.16
Total VFA, mmol	28	97.92	13.24	65.60	112.20
Acetate, %	25	68.15	6.91	49.90	76.10
Propionate, %	25	18.70	3.65	14.00	26.41
Butyrate, %	25	11.78	2.80	7.73	17.23
A/P ratio	16	3.12	0.58	2.27	3.89

*N *number of data points, *Min *minimum observed value, *Max *maximum observed value, *SD *standard of deviation, *DM *dry matter, *OM *organic matter, *CP *crude protein, *NDF* neutral detergent fiber, *ADG *average daily gain, *FCR *feed conversion ratio, *ECM *energy-corrected milk, *BUN *blood urea nitrogen, *VFA *volatile fatty acid, *A/P *acetate/ propionate

Table 3 summarizes the results of the effects of several important covariates that could potentially influence the main production outcomes, including the type of BSF product (BSFM vs. BSFO), inclusion levels, ruminant species, and country of study. Overall, the results showed that the explanatory power of covariates varied across outcomes, with some responses being strongly influenced by specific study characteristics. For ADG, the type of BSF did not statistically influence the outcome (*P* = 0.507). However, ruminant species and country were both significant moderators (*P* < 0.001) for ADG, indicating that differences in growth responses were largely driven by the type of ruminant and the geographical conditions (experimental setting). However, high residual heterogeneity was observed (I² = 93.9%), suggesting the specific-study variance that influences the outcome. The inclusion levels and type of ruminants significantly impacted DMI (*P* < 0.001), but neither type of BSF products (*P* = 0.194) nor country (*P* = 0.415) had a significant effect. For FCR, none of the covariates had a statistical effect (*P* > 0.05). On one hand, DMD significantly differed depending on the BSF inclusion levels (*P* < 0.001) and animal species (*P* = 0.018). Neither BSF type (*P* = 0.395) nor country (*P* = 0.348) influenced DMD outcomes. Publication bias assessment resulted in consistent outcomes based on funnel plot asymmetry test, Begg’s test, and Egger’s test, where no significant bias was observed for ADG, DMI, FCR, and DMD (*P* > 0.05). However, high residual heterogeneity (I² >75%) was observed across variables. The high heterogeneity observed in the present meta-analysis indicates substantial differences among studies in the experimental factors, in addition to the random study-specific variability. The potential sources of heterogeneity include differences in ruminant species (cattle vs. small ruminants), physiological stage, diet composition, forage-to-concentrate ratio, inclusion level, and processing method of BSFM, replacement rate of soybean meal, and the length of the experiment across studies. In addition, variations in environmental conditions, feeding management, and analytical methodologies used across studies may have further contributed to the observed variability. Such heterogeneity is expected in animal nutrition meta-analyses because nutritional responses are strongly influenced by both dietary and management-related factors, as also suggested by previous meta-analyses [41, 42].

**Table 3:** Summary of the effects of dietary black soldier fly and covariates on production performance of ruminants

Covariates	Response variable (*P*-value of covariates)			
	ADG	DMI	FCR	DMD
N	20	39	20	32
Type of BSF	0.950	0.194	0.934	0.395
Levels	0.507	< 0.001	0.611	< 0.001
Animals	< 0.001	< 0.001	0.568	0.018
Country	< 0.001	0.415	0.568	0.348
*I*^2^ and QE	93.9%; <0.001	95.3%; <0.001	80.3%; <0.001	98.7%; <0.001
Begg’s Test	0.869	0.618	0.754	0.278
Egger’s Test	0.553	0.328	0.950	0.234

Random intercept per experiment with REML estimation; k = the number of data points. QM = test of moderators; QE = test of residual heterogeneity

*ADG* average daily gain, *DMI* dry matter intake *FCR* feed conversion ration, *DMD* dry matter digestibility

**Fig. 3:**
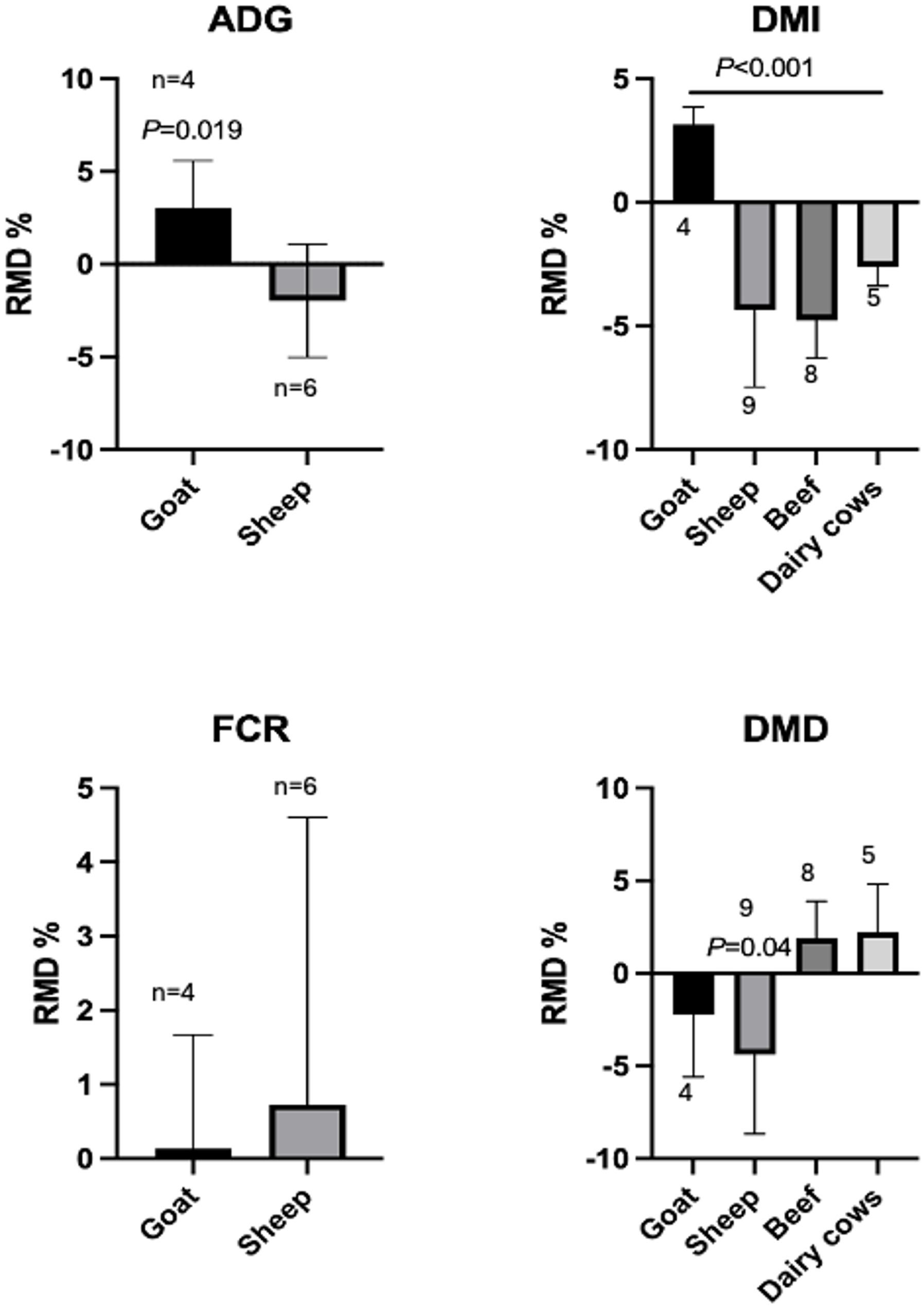
Subgroup meta-analysis of the effect of dietary black soldier fly larvae meal on the main parameters based on type of animals. Value in each bar represents the number of comparisons

Results of random-effect models meta-analysis on all measured parameters are shown in Table 4. The dietary inclusion of BSF did not affect DMI (RMD = − 1.81%; *P* = 0.121; *n* = 39). However, subgroup meta-analysis revealed different responses depending on ruminant species (*P* < 0.01). Specifically, DMI was higher in goats under BSF feeding (*n* = 4), while sheep (*n* = 9), beef cattle (*n* = 8), and dairy cows (*n* = 5) exhibited reductions in DMI, with beef (-4.76%) and dairy cows (-2.61%) showing the largest decrease (Fig. 3). The effect on NDFI was not significant, but BSF inclusion decreased CPI (RMD = − 3.54%; 95% CI: − 6.04 to − 1.03; *P* = 0.006; *n* = 19). While no significant effects were observed for CPD and NDFD, species-specific effects were observed on DMD (*P* = 0.04). In beef cattle (+ 1.89%; *P* = 0.06; *n* = 8) and dairy cows (2.53%; *P* = 0.08; *n* = 5), DMD tended to be higher under BSF feeding, while no difference was observed in goats and sheep. No significant effects were detected for (ADG, FCR, milk yield, milk yield/DMI, or energy-corrected milk (ECM), suggesting that BSF-based diets support comparable performance to conventional rations across diverse ruminant species. However, species-specific results were found where the ADG (RMD = 3.03%; *P* = 0.019; *n* = 4) improved in goats, but sheep and cattle were similar compared to the control. Moreover, interesting results were observed on the improved effects on ECM/DMI (3.33%; *P* = 0.024; *n* = 3) and milk fat percentage **(**RMD = + 3.16%; *P* = 0.001; *n* = 8), highlighting a positive response in milk fat synthesis when BSF was dietarily incorporated. Other milk components (milk protein and lactose) were not significantly influenced.

**Table 4:** Summary of the results of meta-analysis

Items	*N*	Estimate (RMD%; 95% CI)				*P*-value	Heterogeneity	
		RMD %	SE	Lb	Ub		I^2^	Q
Feed intake (kg/d) and digestibility (%)								
DMI, kg/d	39	-1.811	1.168	-4.099	0.479	0.121	93.03	< 0.001
CPI, kg/d	19	-3.536	1.278	-6.042	-1.031	0.006	78.29	< 0.001
NDFI, kg/d	17	-1.024	2.009	-4.962	2.915	0.610	87.47	< 0.001
DMD, %	32	0.794	0.791	-0.756	2.344	0.315	98.20	< 0.001
CPD, %	25	1.056	1.559	-2.002	4.112	0.498	97.39	< 0.001
NDFD, %	32	0.303	1.011	-1.678	2.284	0.764	92.74	< 0.001
Production performance								
ADG, kg/d	20	0.414	2.253	-4.000	4.829	0.854	85.65	< 0.001
FCR	20	-0.147	0.525	-3.136	2.841	0.923	74.31	0.001
Milk yield, kg	8	1.426	2.413	-3.304	6.156	0.555	89.63	< 0.001
Milk yield/ DMI	3	0.373	1.119	-2.567	1.821	0.739	71.39	0.145
ECM, kg	5	2.511	1.642	-0.706	5.729	0.126	66.53	0.051
ECM/ DMI	3	3.333	1.481	0.429	6.237	0.024	1.00	< 0.001
Milk fat, %	8	3.156	0.859	1.472	4.841	0.001	65.01	0.022
Milk protein, %	8	0.138	0.932	-1.689	1.964	0.883	92.84	< 0.001
Milk lactose, %	8	0.869	0.761	-0.621	2.360	0.253	91.55	< 0.001
Rumen fermentation								
N-NH_3_, mg/dL	4	-3.333	4.049	-11.980	5.309	0.449	79.78	0.007
Protozoa	8	-5.143	7.800	-20.430	10.140	0.509	91.06	< 0.001
pH	24	-0.624	0.466	-1.537	0.288	0.180	95.97	< 0.001
Total VFA, mM	28	7.689	3.413	1.001	14.380	0.024	97.01	< 0.001
Acetate, %	25	-0.153	0.653	-1.433	1.128	0.815	95.77	< 0.001
Propionate, %	25	0.183	1.548	-2.853	3.217	0.906	96.05	< 0.001
Butyrate, %	25	-3.558	2.123	-7.719	0.604	0.094	92.40	< 0.001
A/P ratio	16	4.314	4.078	-3.368	12.310	0.291	94.17	< 0.001

*RMD* raw mean difference, *SE* Standard error, *Lb* lower bound, *Ub* upper bound, *I*^2^ heterogeneity value, *Q p*-value of the heterogeneity index

Overall, our current meta-analysis identified species-specific responses to dietary BSF supplementation among ruminants. Notably, an increase in ADG was observed in goats, whereas no significant effects were observed in lambs and sheep. Similarly, in beef cattle, BSF supplementation did not influence ADG. These variable outcomes may be attributed to study-specific conditions rather than the effects of BSF. Lu et al. [31] documented higher ADG in goats receiving a diet with 5% BSFM, intended to partially substitute soybean meal. The increased ADG in their study was associated with higher DMI in the 5% BSFM group, potentially providing a greater nutrient supply, particularly amino acids, to the animals. In contrast, Odeon et al. [28] reported that lambs exhibited no significant change in ADG, despite DMI not being affected, suggesting a comparable nutrient supply and thus no increase in ADG. The above two studies suggest that the higher ADG was mainly attributed to higher DMI rather than BSFM alone. The different responses of goats and lambs could be attributed to the different physiological stages (age) in which the palatability of the BSMF is lower in younger animals (lambs).

In cattle, although dietary BSFM reduced the DMI in both beef and dairy cattle, the production performance remained unaffected. These reductions may indicate palatability differences or the effects of dietary formulation when BSF ingredients partially replace SBM protein sources in larger ruminants. In the studies included in this meta-analysis, Fukuda et al. [15] and Carrasco & Drewery [9] reported inconsistent findings. Fukuda et al. [15] observed a stimulation in feed intake, whereas Carrasco & Drewery [9] noted a reduced feed intake in ruminants fed with BSF-containing concentrate compared to conventional SBM. Although these differences were attributed to the use of defatted versus non-defatted BSF, several nutritional factors may also explain the contrasting responses. Non-defatted BSF generally contains substantially higher fat levels, particularly medium-chain fatty acids such as lauric acid [1, 2], which may negatively affect rumen fermentation (e.g., suppress fiber-degrading bacteria) and reduce palatability when included at higher dietary concentrations. In contrast, defatted BSF meal has a higher CP concentration and lower lipid content, which may improve rumen compatibility and nutrient utilization without imposing excessive fat load on the rumen ecosystem. However, in dairy cows, inclusion of defatted BSF resulted in reduced DMI [30] when it was used to fully replace SBM at a 9% DM inclusion rate. The reduced DMI in dairy cows could be attributed to the difference in NDF content between insect-based (BSF) and plant-based (SBM) sources, potentially affecting diet passage rate. Another factor contributing to the lower DMI of the BSF-based diet could be the FA profile and other nutrient components, which might have influenced the palatability and feed utilization by the animals. For instance, BSF was reported to contain a higher lauric acid content than SBM (17.5% vs. 3.0%), and lauric acid has been reported to decrease DMI in other studies [43, 44]. In this meta-analysis, lower CP intake was generally associated with reduced DMI in cases where lower DMI was reported [9, 30].

In addition, BSF is recognized for its substantial chitin content. Chitin, N-acetyl-d-glucosamine (GlcNAc), (1–4)-linked 2-acetamido-2-deoxy-β-d-glucan homopolymer, constitutes a component of the BSF larvae exoskeleton and is known to be indigestible in the rumen [45] due to the absence of the chitinase enzyme. It naturally forms a matrix with other nutrients, such as lipids and proteins [46], which may decrease nitrogen availability for the rumen microbes, thereby diminishing microbial protein synthesis and amino acid supply. Consequently, a high inclusion of BSF, particularly when the chitin content is elevated, can lead to reduced nutrient utilization and disrupt ruminal production performance. However, our findings indicated that DMD, CPD, and NDFD were not influenced by BSF inclusion, and production performance, particularly in beef and dairy cows, remained unaffected. This may be attributed to the low chitin content, which may not be sufficient to impede nutrient digestion. Supporting this, a previous study demonstrated that chitin contributed only up to 6.9% nitrogen [47], which may not be biologically significant with low BSF inclusion. Conversely, chitin is advantageous when administered at low levels because of its anti-inflammatory and antimicrobial properties [48, 49]. Chitin and chitosan have been shown to perform multiple biological roles as immunomodulators, antioxidants, and antibacterials, which can enhance nutrient digestion and ruminant production efficiency [50, 51]. A recent report supports the potential for improved nutrient digestion due to the observed activity of chitinolytic bacteria in the rumen [52]. In contrast, digested chitin contributes to increased nitrogen availability. The improved nutrient digestibility was particularly evident in this meta-analysis, where DMD was higher in beef and dairy cattle fed diets containing BSF. The enhanced digestibility in cattle may reflect better adaptation to lipid-rich or chitin-containing feed ingredients than in small ruminants. However, the specific mechanism and precise contribution of chitin in the rumen warrant further investigation.

Milk production was not affected by BSF inclusion in this meta-analysis, but contrasting results have been reported in the literature. Braamhaar et al. [30] found no effect on milk yield by including 10% BSF in the diets despite a lower CP intake. This unaffected milk yield might be due to the higher NDF digestibility (NDFD) observed in their study, as well as the plausible beneficial effects of chitin, as explained above. The higher NDFD might be attributed to the physical characteristics of BSF, where the mechanical processing of BSF aids in improving the physical structure of BSF to be more accessible to rumen microbes. The improved NDFD would have resulted in greater VFA production, which is essential for energy supply for the animals; however, VFA was not measured in studies utilizing BSF in dairy cows. Higher total VFA production and propionate proportion were reported in an in vitro study with 5% BSF inclusion [31, 33], which could partly explain the higher energy supply when BSF was included, thus maintaining milk yield despite the lower CP intake. In contrast, higher milk yield and ECM were reported in another in vivo study using BSF oil, despite the unaffected nutrient digestibility value and similar energy intake [32]. As this study utilized isocaloric and isonitrogenous diets, the increased milk yield might not be related to energy supply and rumen fermentation efficiency, as the latter was not reported. The only variable that could plausibly explain the higher milk yield was the enhanced antioxidant activity due to BSF oil inclusion [43]. However, the difference in FA composition between BSF and palm oil could not be ruled out because the former is known to have greater lauric acid content. Lauric acid, which has shorter carbon chains, is easier to digest in the rumen; thus, a higher VFA level was found in dairy cows fed the BSF oil diet. Higher VFA production and greater milk yield were reported when BSF was supplemented at 100 g/d [13].

Recent findings indicate that BSF supplementation enhances milk fat synthesis, a crucial aspect of dairy quality, without negatively affecting the FCR or ADG [9, 15]. For example, BSF feeding has been linked to an increased milk fat percentage, as evidenced by studies reporting superior fat-to-protein ratios (approximately 1.09–1.1) in cows fed BSF compared with those receiving conventional feeds [44]. The primary factor driving these improvements is the lipid composition of BSF, particularly its MCFA and SFA, such as lauric acid and myristic acid. These MCFA bypass ruminal biohydrogenation and are directly absorbed into the bloodstream, where they contribute to milk fat synthesis in the mammary glands [9]. Moreover, these fatty acids, specifically lauric and myristic acids, suppress methanogenesis, thereby reducing greenhouse gas emissions and enhancing nutrient utilization and feed efficiency in ruminants. Yanza et al. [53] have confirmed that MCFA, even when derived from plant sources, can significantly influence rumen fermentation and production performance in ruminants, affecting milk production and weight gain. The combination of lauric acid and myristic acid can modify ruminal fermentation by altering microbial populations and improving fermentation efficiency, which is indirectly related to ruminant production performance, including milk yield and nutrient content [53]. These findings suggest that BSF, with its inherent MCFA content, could similarly enhance fermentation efficiency and nutrient utilization, leading to favorable outcomes for milk fat synthesis and overall animal productivity in dairy cows fed BSF.

In addition to its lipid profile, BSF inclusion appears to enhance rumen fermentation efficiency, as evidenced by the significant increase in total VFA in animals fed BSF [54]. Although acetate production did not significantly increase, the overall increase in VFA concentration suggests an improvement in fermentation efficiency, thereby providing more substrates for milk fat synthesis. This process occurs in the mammary glands, where substrates such as acetate and butyrate are utilized for de novo fatty acid synthesis in the mammary glands. Studies have demonstrated that feeding BSF improved nutritional parameters crucial for managing metabolic challenges in high-producing dairy cows [13, 31]. Moreover, the bioactive components of BSF, including MCFA and chitin, modulated ruminal microbial activity. The MCFAs selectively inhibit gram-positive bacteria, such as *Streptococcus bovis* and *Clostridium spp*., which are associated with inefficient fermentation [55]. By altering the microbial community, BSF promotes more efficient fermentation, leading to greater VFA production and enhanced feed efficiency, ultimately supporting the synthesis of milk fat.

The findings also demonstrate species-specific response patterns. For example, dairy cows exhibited a significant increase in milk fat percentage, a response not observed in goats and sheep, likely attributable to differences in metabolic pathways and ruminal fermentation among species. In dairy cows, the increase in milk fat, coupled with an improved energy-corrected milk (ECM) to DMI ratio, underscores the potential of BSF to enhance both milk quality and feed efficiency. Research has indicated that the inclusion of dietary fat, particularly from BSF, enhances VFA concentration [34]. An increase in SCFA production contributes to better feed efficiency, as reflected by an improved feed efficiency (ECM/DMI) in dairy cows supplemented with BSF. Furthermore, BSF inclusion was associated with reduced methane emissions, suggesting that BSF improves rumen fermentation efficiency and is a sustainable feed source. This highlights the potential of BSF not only to enhance milk fat but also to mitigate the environmental impact of dairy farming by reducing greenhouse gas emissions [56]. Thus, BSF serves as an effective and sustainable feed ingredient for improving milk fat content and dairy farm sustainability.

### Black soldier fly alters rumen metabolism

**Fig. 4:**
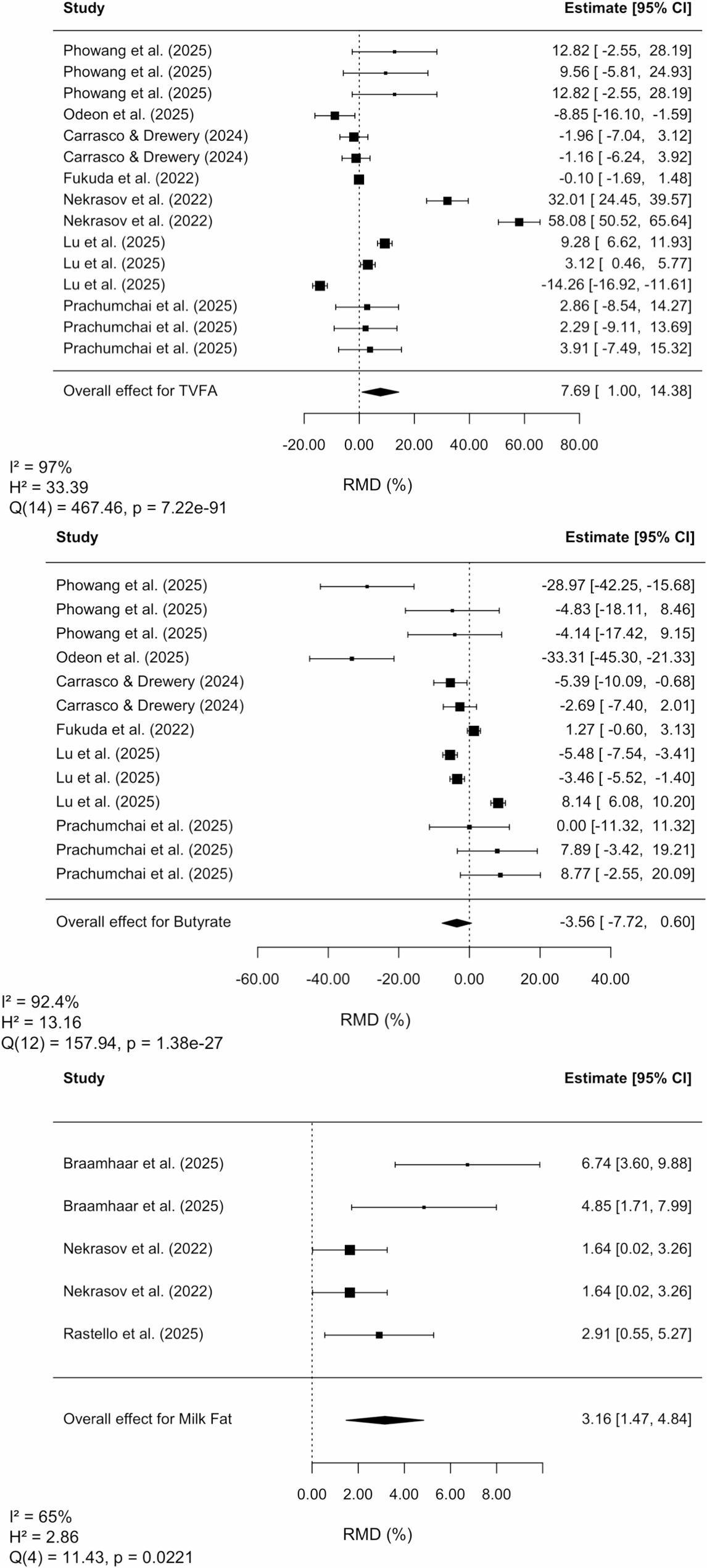
Forest plot summarizing of the effect of dietary black soldier fly larvae meal on ruminal total VFA, butyric acid, and milk fat content

Results of the rumen fermentation profile indicated notable shifts with dietary BSF inclusion. Total VFA concentration was higher with BSF inclusion (RMD = 7.69%; *P* = 0.024; Fig. 4; *n* = 28) while butyrate proportion tended to be lower (RMD = − 3.56%; *P* = 0.094; *n* = 25). However, the VFA data were only reported by studies involving beef cattle and sheep without reporting results in dairy cows. Other rumen parameters, including NH_3_, protozoa count, rumen pH, acetate, propionate, and A/P ratio, were not affected by BSF inclusion. The notable increase in the total VFA concentration suggests that BSF enhances rumen fermentation efficiency. Enhanced VFA production is generally linked to increased energy availability in ruminants, supporting improved feed conversion and overall productivity [15, 57]. The increase in VFA concentration also reflects a positive modulation of rumen microbial activity, highlighting the potential of BSFM to increase the bioavailability of energy from the diet.

However, the proportion of butyrate, a key VFA associated with the health of the rumen epithelium and gut function, tended to decrease with BSF inclusion. Butyrate serves as a crucial energy source for colonocytes in the rumen and plays a key role in maintaining gastrointestinal tract integrity [13]. It is possible that MCFA and chitin, which are key bioactive components of BSF, selectively modulate the microbial populations responsible for butyrate production. Lauric acid, as the primary MCFA content, and chitin both exert antimicrobial and microbiota-modulating effects in the rumen, as supported by prior studies demonstrating that BSF-derived MCFA and chitin selectively decreased butyrate-related taxa and overall rumen microbial communities, resulting in partial shift of VFA production [31,59]. This microbial shift could explain the reduction in the butyrate proportion, although its impact on rumen epithelial health remains uncertain.

Despite the tendency for lower butyrate, other important rumen parameters, such as NH₃ concentration, protozoal count, rumen pH, and A/P ratio, remained unaffected by BSF inclusion. This stability suggests that BSF inclusion does not drastically alter the rumen ecosystem. The unchanged A/P ratio is particularly noteworthy, as it suggests that BSF does not significantly shift fermentation pathways that are typical of forage-based diets, where acetate remains the dominant VFA [31]. A stable rumen pH further supports the idea that BSF inclusion does not induce acidosis or disrupt the ruminal environment, both of which are critical for maintaining microbial balance and efficient fermentation. These findings are consistent with those of previous studies showing that BSF can be included in ruminant diets without inducing adverse changes in ruminal pH, a key factor in rumen health and overall metabolic function [58].

Interestingly, the absence of significant changes in other rumen parameters, such as protozoal count, supports the notion that bioactive compounds of BSF do not disturb the balance of ruminal microbial populations. This absence effect might also be attributed to the fact that defatted BSF meal does not exert antimicrobial properties. In studies reporting lower protozoal count and microbial modulatory effect, previous authors suggest that such effect was due to the non-defatted BSF or BSF oil rich in MCFA [15,53,60]. Therefore, minimal effect of BSF meal on protozoal population in the present meta-analysis suggests that BSF supplementation can enhance fermentation without negatively disrupting rumen microbiota.

## Conclusions

This meta-analysis elucidates the beneficial effects of BSF on rumen fermentation and milk fat synthesis in dairy ruminants due to an increase in total VFA concentration, indicating enhanced fermentation efficiency. Notably, improvements in milk fat percentage and ECM efficiency were observed, suggesting that BSF contributes to the higher energy provision for milk fat synthesis. However, a reduction in the butyrate proportion was noted, which may impact gut health and energy metabolism, necessitating further investigation. Species-specific responses were evident, with goats exhibiting the most favorable production effects, whereas sheep did not show significant changes. This variability warrants further species-specific feeding strategies, especially due to the limited number of in vivo studies (12 in vivo trials) so far. Additionally, long-term effects and outcomes related to carcass quality remain underreported. Despite the decrease in butyrate, the stability of other ruminal parameters (e.g., pH and A/P ratio) suggests that BSF does not disrupt the rumen ecosystem. The bioactive components of BSF, such as medium-chain fatty acids and chitin, likely modulate microbial populations and enhance fermentation efficiency without causing major disruption. Future research should address these gaps, particularly in dairy cows and long-term studies, to better understand the role of BSF in ruminal health, nutrient utilization, and sustainable livestock production.

## Data Availability

Data available on request from the authors.
